# The adequacy of lymph node harvest in concomitant neck block dissection and submental island flap reconstruction for oral squamous cell carcinoma; a case series from a single Egyptian institution

**DOI:** 10.1186/s12903-015-0064-0

**Published:** 2015-07-14

**Authors:** Islam A. Elzahaby, Sameh Roshdy, Fayez Shahatto, Osama Hussein

**Affiliations:** Mansoura University cancer Center, Mansoura University, Mansoura, 35516 Egypt

**Keywords:** Oral squamous cell carcinoma, Neck block dissection, Submental flap

## Abstract

**Background:**

Squamous cell carcinoma (SCC) is a fairly common tumor of the oral cavity. This tumor may affect any part of the mucosa of the oral cavity especially the tongue, the floor of the mouth and lips. The encountered intra-oral defects after tumor resection are often large and require climbing up the reconstruction ladder to more complex reconstructive options for accepted functional and cosmetic results to be achieved. However, most of the patients are old with medical co-morbidities requiring fast, simple, less morbid reconstructive option such as local flaps. The myocutaneous submental island flap has emerged as a simple and fast reconstructive technique that provides thin, pliable tissue with adequate volume and reliable blood supply. However, one major concern regarding the utility of the submental flap for repair of post-ablative tumor defects is the presumed interference with adequate lymph node neck dissection.

**Methods:**

In this study, we present a cohort of thirty-six consecutive patients who were operated for oral SCC. All patients were offered submental island flap reconstruction of their resultant defects together with ipsilateral selective neck block dissection of levels I, II, III and IV; and the nodal yield of each level was tested pathologically.

**Results:**

Nodal harvest was ≥12 in 88 % of the patients. Complications were encountered in two patients (5.5 %).

**Conclusion:**

Our data suggest that adequate cervical lymph nodes dissection, specifically level I and II cervical lymph nodes, can be fulfilled with concomitant submental island flap elevation.

## Background

Squamous cell carcinoma (SCC) represents over 90 % of oral malignant lesions, and about 4.500 people will be diagnosed with oral cancer every year in Egypt [1]. This tumor may affect the mucosa of the floor of the mouth, cheek, tongue and /or inner lip surface [2]. Reconstruction of defects resulting from extirpation of these tumors is often challenging due to the multifaceted aspects of functional and cosmetic considerations [3]. Patients with oral mucosal carcinomas are commonly old chronic smokers with co-morbidities [4]. Thus several local flap options were implemented for rapid, less morbid reconstructive procedure. The myocutaneous submental island flap has emerged as a simple reconstructive technique that provides thin, pliable tissue with adequate volume and reliable blood supply [5–7]. However, the submental island flap is overlying the main lymph node basin for all intra- oral malignancy which is level I and II cervical LNs making its elevation relatively difficult and claimed to affect its oncological safety. In this study, we present a cohort of consecutive thirty-six patients who were operated for oral SCC. All patients were offered selective neck block dissection which gave us the chance to objectively test the adequacy of neck dissection with concomitant submental flap elevation using the parameter of pathological final nodal staging.

## Methods

**Fig. 1:**
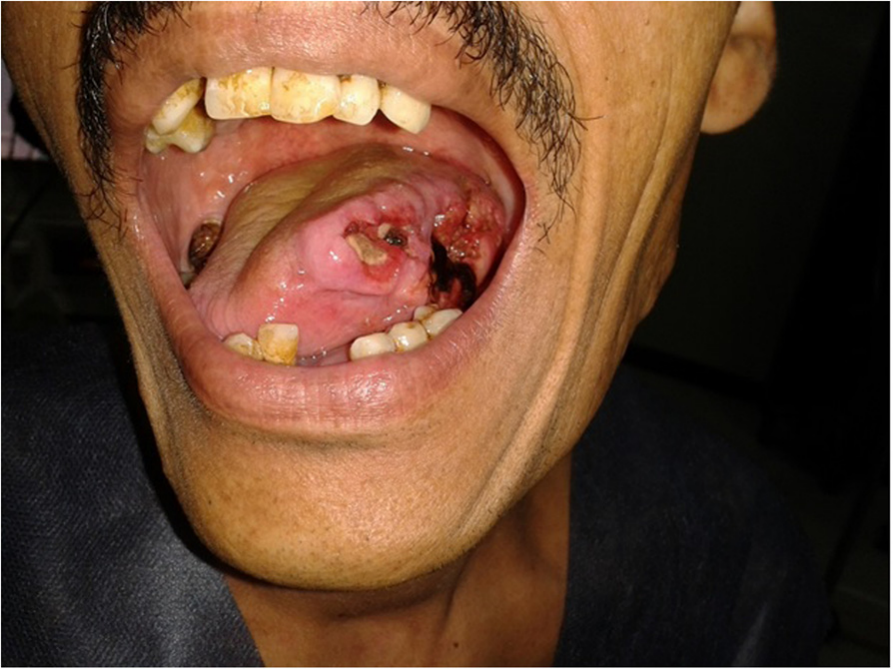
Preoperative view of large left sided tongue squamous cell carcinoma

**Fig. 2:**
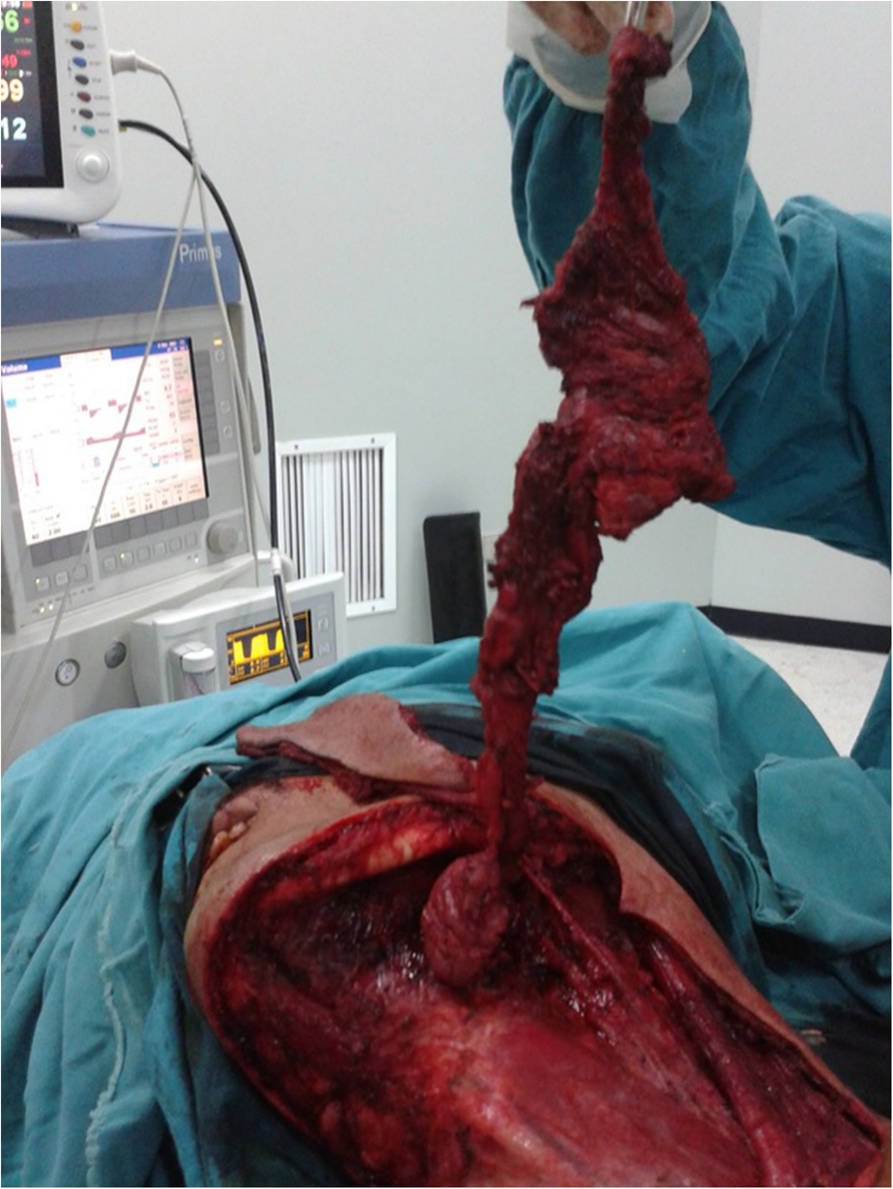
Intraoperative view of hemi-glossectomy with cervical lymph node en-bloc resection (compartemental tongue resection) after submental flap harvest

This is a retrospective single institution study including all consecutive patients with intraoral T_2_ through T_4a_ SCC presented to the authors’ service at the surgical oncology unit at Mansoura University from September 2009 to August 2013. Written informed consent was obtained from all patients of the study for the procedure, photographing and publishing in print and electronic form. This is a retrospective study and ethics board review was not required. Data for thirty-six patients were available for inclusion in this study. Patients with mandibular invasion, clinical nodal status beyond N_1_ or with distant metastases were excluded. Preoperative investigations included histopathological diagnosis and CT staging of the tumor extent. All patients were operated for simultaneous tumor resection and reconstruction of their intraoral defects with orthograde submental island flap. After tumor wide excision and confirmation of adequate safety margin with frozen section examination, reconstruction of the mucosal defect began with elevation of the submental flap island pedicled on the submental artery. The skin island extended from one angle of the mandible to the other, and ipsilateral anterior belly of digastric was included in the flap in all cases, Doppler localization of the pedicle was not needed in any of the cases. Primary closure of the donor site was done with undermining of the lower neck flap at the subcutaneous plane superficial to the platysma. All cases had selective level I-IV nodal neck dissection which was performed after flap elevation (Fig. 1, 2). Nodal yield of each level was counted and number of pathologically involved LNs was stated. No tracheostomy was needed except for one case with total glossectomy. pN+ patients received postoperative radiotherapy of 66–70 Gy. Neoadjuvant therapy was not used in this study.

## Results

This study included thirty-six patients with primary intraoral SCC. Baseline criteria of the patients are presented in Table 1.

**Table 1:** Base line criteria of the patients of the study

PATIENTS’ AGE	MEDIAN	61
	RANGE	24 − 72
GENDER	MALE	28
	FEMALE	8
TUMOR STAGE	I	0
	II	13
	III	14
	IVA	9
TUMOR GRADE	I	20
	II	16
	III	0

**Fig. 3:**
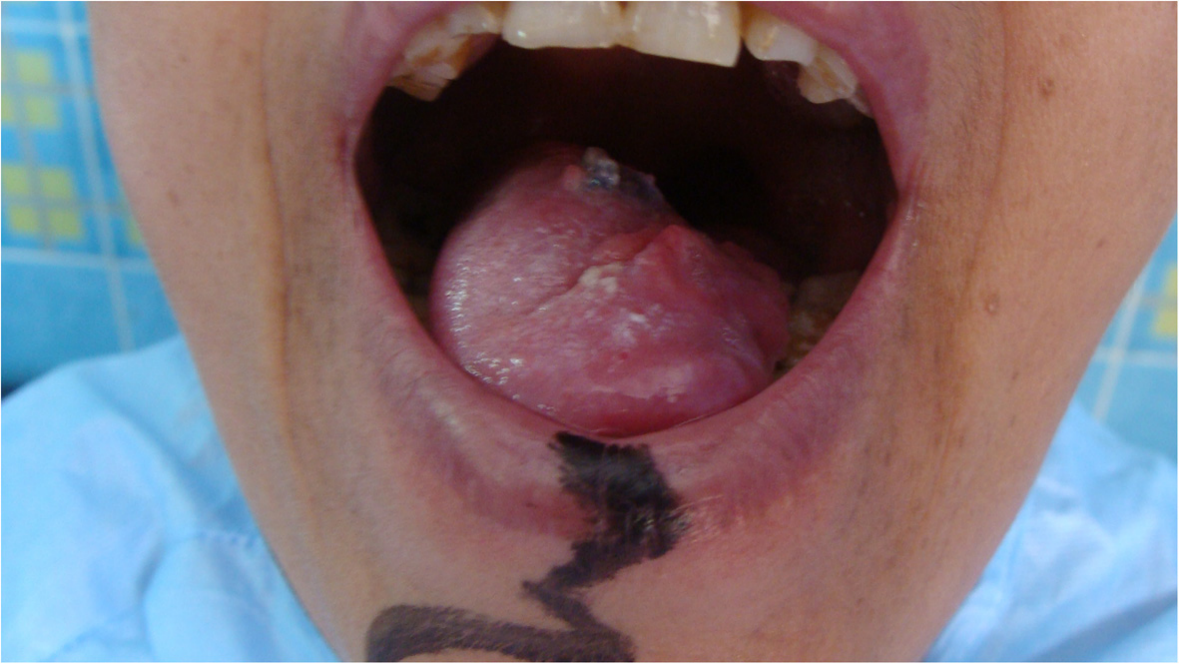
Preoperative view of female with squamous cell carcinoma of the whole tongue

**Fig. 4:**
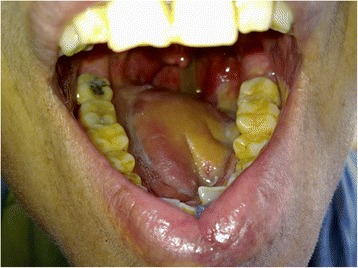
Seven months postoperative view of the total glossectomy patient

Eleven patients had inner cheek defects, four other patients had full thickness cheek defects involving the angle of the mouth with adjoining parts of the upper and lower lips, six patients had anterior mouth floor defects, fourteen patients had combined defect of the hemi-tongue and underlying floor of mouth and one patient had total tongue defect with floor of the mouth (Fig. 3, 4). The area of the defect varied from 4 to 10 cm^2^. Twenty patients were clinically node negative and sixteen patient had clinically significant lymph nodes in the submental and/or submandibular region. One incident of partial flap necrosis occurred in the case of total glossectomy. No complete flap necrosis occurred. The average time required for the reconstruction was around 2 h.

Two patients developed salivary leak, one with total glossectomy defect and the other with compartmental tongue resection defect. Salivary leak stopped at day 28 and day 22 respectively. All other patients had smooth postoperative course and early hospital discharge after 10 days. The postoperative course after total glossectomy (single patient) was distinctive. In this patient, tube feeding was required and repeated chocking was observed in the first two months after surgery. No donor site morbidity occurred in any of the cases of the study. The structural and functional outcome in all cases was acceptable except for the problem of intraoral hair in-growth within the mouth in male patients but was effectively managed with repeated mechanical or laser depilation sessions.

All patients received ipsilateral selective neck dissection from level I- IV. Pathological examination of the nodal yield for each level was done, a median nodal harvest of 16 nodes (range 11–33) (Table 2). Thirty two patients (88.8 %) had at least 12 nodes identified in the pathological tissue specimen.Twelve patients (33.3 %) had pathological nodal invasion including a single patient with extracapsular extension.

**Table 2:** The range of total and compartmental (level I&IIA) cervical lymph node (LN) yield in the patients of the study

clinical nodal stage	Number of patients	Range of total LN harvest	Range of LN yield in level I& level IIA	Range of pathologically positive LN in level I & level IIA	Number of patients with Pathologically positive nodes (pN+)	
N0	20	11 − 24	6 − 12	0 − 1	pN0 19	19
					pN1(mi)	1
N1	16	12 − 33	8 − 17	1 − 7	pN0	4
					pN1	3
					pN2a	3
					pN2b	6

The follow up period ranged from 11 months to 48 months. The primary outcome measures were morbidity and recurrence of the disease.

None of our patients developed regional nodal recurrence. However, three of our patients developed local recurrence (8.3 %), all of them were young patients whose ages were 26, 30, 18 years respectively, the former two patients were non-smoker, with occupational exposure to hydrocarbones (mechanic) and wood dust (carpenter) respectively, the third patient was a young female with xeroderma pigmentosa. In the first two patients the primary cancer was in one side of the tongue, the tumor relapsed within the first year at the base and the other side of the tongue suggesting a second primary due to field cancerization rather than recurrence. In the patient with xeroderma pigmentosa, the primary cancer was a full thickness cheek cancer, and the recurrence occurred one year later which was on the outer cutaneous side of the cheek together with nearby multiple exophytic malignant growths on the lips, nose and chin which are consistent with the natural course of this genetic disease. All these recurrence were treated by re-surgery.

## Discussion

In this study, we present the early reconstructive and oncological outcome of thirty-six consecutive patients with oral SCC treated with radical excision, selective block neck dissection and myocutaneous submental island flap reconstruction. We examined the final pathological nodal status and we objectively evaluated the effect of using this reconstructive technique on the adequacy of lymph node dissection. To our knowledge, this is the first report to provide clear, objective indication of adequate neck dissection with concomitant submental flap elevation in cases with SCC of the oral cavity.

Free tissue transfer has been the primary option for reconstruction of post-ablative tissue defects in oral cancer patients. Free flaps provide generous donor tissue volume with adequate blood supply for most cases [8, 9]. The technique may not be preferred in vessel-depleted irradiated neck, morbid patients who cannot tolerate prolonged anesthesia or in communities where the extra cost of microvascular surgery is prohibitive [10, 11]. Pedicled myocutaneous flaps such as the pectoralis major flap are classic option characterized by technical simplicity and reliable blood supply [12, 13]. These flaps are often too bulky, necessitate secondary revisional surgeries and had higher rate of complications especially in female patients [14, 15].

Several local fasciocutaneous and myocutaneous flaps have been designed to provide thin coverage with good tissue colour and texture match. Of these flaps, the submental artery flap is a viable option with many potential advantages [7, 16]. The flap can be used for reconstruction of defects in the lower and mid thirds of the face and of the oral cavity. It provides skin quality with good matching with the recipient area. The available skin island extends as far as the contralateral angle of the mandible due to the rich anastmosis across the midline and through the underlying platysma [11, 17]. The flap has an adequate arch of rotation which can be even increased by dividing the facial vessels proximal to the submental artery origin and raising the flap based on retrograde flow from the distal facial artery [5, 18] or even used as a free flap [19]. The submental flap can be raised as fasciocutaneous, myocutaneous, osteomuscular and even with inclusion of the ipsilateral submandibular gland, these structural variants add to the versatility of reconstruction [20, 21]. It can be an excellent choice in patients with limited physiologic reserve when operative trauma and delayed postoperative recovery are major concerns. The use of this flap however was halted due to concerns about interference of flap elevation with sound nodal dissection of the neck specifically level 1 and 2a.

Amin and colleagues investigated the problem and these authors recommended the completion of the neck dissection before any attempts to harvest the flap [22].

Also, Xuwei et al. recommended abandoning the use of the submental flap if the submental lymph nodes were enlarged [21].

Recent report by Hayden group examined a series of fifty patients with stage I-III malignancy of the oral cavity. Selective neck dissection of level 1 only (the submental and submandibular nodes) was performed. Occult nodal infiltration was 10 %. All patients in this series were clinically node negative [23].

Pathological examination of the neck block dissection remains the only objective evidence of adequate neck node staging. Ten or more lymph nodes are required for satisfactory examination of the pathological specimen and designing accurate pN stage [24]. In the cadaveric study of Friedman et al. [25], the average number of lymph nodes harvested from levels I-V was twenty-four, thirteen nodes for levels I-III and nineteen for levels II-IV. In our study the total median cervical lymph node yield was 16 (range 11–24) for cN0 patients and was 25 (range 12–33) for cN1 patients. All patients received selective neck dissection as per current clinical guidelines [26]. Selective neck dissection is known to offer comparable oncologic results with less morbidity than radical neck dissection [27]. Thus our series provides an opportunity to draw an objective conclusion about the question of the adequacy of nodal dissection in patients who had concomitant submental flap reconstruction. We have shown that all of our patients had adequate nodal staging as per AJCC recommendation.

## Conclusion

The submental island flap is a highly recommended flap for reconstruction of different site and size oral cavity defects. Besides being easy, reliable, not bulky, with good arc of rotation and without donor site morbidity, it doesn’t interfere with sound cervical lymph node dissection and is oncologically safe in patients with N0-N1 disease.
